# An Enhanced Full-Form Model-Free Adaptive Controller for SISO Discrete-Time Nonlinear Systems

**DOI:** 10.3390/e24020163

**Published:** 2022-01-21

**Authors:** Ye Yang, Chen Chen, Jiangang Lu

**Affiliations:** 1 State Key Laboratory of Industrial Control Technology, College of Control Science and Engineering, Zhejiang University, Hangzhou 310027, China; yyang0922@zju.edu.cn (Y.Y.); cchen618@zju.edu.cn (C.C.); 2 Zhejiang Laboratory, Hangzhou 311121, China

**Keywords:** SISO discrete-time nonlinear systems, full-form model-free adaptive controller, fuzzy neural networks, long short-term memory neural networks, three-tank system

## Abstract

This study focuses on the full-form model-free adaptive controller (FFMFAC) for SISO discrete-time nonlinear systems, and proposes enhanced FFMFAC. The proposed technique design incorporates long short-term memory neural networks (LSTMs) and fuzzy neural networks (FNNs). To be more precise, LSTMs are utilized to adjust vital parameters of the FFMFAC online. Additionally, due to the high nonlinear approximation capabilities of FNNs, pseudo gradient (PG) values of the controller are estimated online. EFFMFAC is characterized by utilizing the measured I/O data for the online training of all introduced neural networks and does not involve offline training and specific models of the controlled system. Finally, the rationality and superiority are verified by two simulations and a supporting ablation analysis. Five individual performance indices are given, and the experimental findings show that EFFMFAC outperforms all other methods. Especially compared with the FFMFAC, EFFMFAC reduces the RMSE by 21.69% and 11.21%, respectively, proving it to be applicable for SISO discrete-time nonlinear systems.

## 1. Introduction

Science and technology advancements have brought significant changes in the industry in recent decades [1], during which many traditional industries have gradually increased the control requirements for production systems, and the majority of current industrial processes are multivariable, nonlinear, strongly coupled and have many operating conditions [2]. The traditional continuous-time control theory is confronted by significant challenges. Powered by computer control theory and technical application, the control of most complex systems can be transformed into the control problems of discrete-time nonlinear systems [3]. In practice, there are many nonlinearities in many application fields, such as robots, process control, biomedical engineering and power systems [4]. In the case of weakly nonlinear systems, the system model can be Taylor expanded near the operating point, and the linear control theory can be introduced to design the controller [5]. However, when the system has model uncertainty caused by the dynamic mutation of the controlled system due to changes in the operating environment, component aging damage or external interference, it is difficult for traditional linear controllers based on fixed parameters to obtain satisfactory control performance.

Nowadays, in the industrial production process, a significant amount of online or offline industrial data comprising factual information on nonlinear systems can be generated, collected and stored [6]. Meanwhile, these data can be analyzed online with the assistance of advanced hardware and software technology. As a result, the direct management of controlled system data to control discrete-time nonlinear systems has become a subject of concern and research. Data-driven control (DDC) [7] is a control method in which the controller is designed entirely from the online and offline I/O data of the controlled system, rather than on precise mathematical models, and thus guarantees the controlled system’s stability, convergence and robustness under certain assumptions. This method avoids restrictions associated with model-based control methods by not relying on the mathematical model of the controlled system when developing the controller and instead just uses the controlled system’s I/O data to identify and optimize the controlled object.

Since the 1990s, a variety of data-driven control methods have emerged in the control research direction. Unfalsified control (UC) [8] uses recursive perjury to select the controller that meets specific performance requirements from the set of candidate controllers as the current controller. Simultaneous perturbation stochastic approximation (SPSA) [9] designs a control performance index function with controller parameters as optimized variables, and uses the system’s I/O data each time to minimize the performance index function to obtain the optimal controller parameters, thereby realizing the design of the controller. Virtual reference feedback tuning (VRFT) [10] utilizes the measured data of the controlled object to convert the controller design problem into a controller parameter identification problem by employing a virtual reference signal. Iterative learning control (ILC) [11] uses the system output error and control input signal of the previous cycle to construct the control input signal of the current cycle to obtain a better control performance than the previous cycle. Lazy learning (LL) [12] uses historical data to establish a local linear model of the controlled system online, and then design a controller with the local linear model at each moment. These DDC methods are now widely employed in practice after years of research. As a DDC method, MFAC [13] employs the dynamic linearization method to develop the equivalent dynamic linearization data model [14] of the controlled system at each sampling time, and then estimates the pseudo partial derivative (PPD) values or pseudo gradient (PG) values [15] to approximate the dynamics of the controlled system. According to the type of dynamic linearization data model [7], MFAC methods can be broadly classified into three types, namely compact-form MFAC (CFMFAC), partial-form MFAC (PFMFAC) and full-form MFAC (FFMFAC). Compared with ordinary DDC methods, MFAC has the following advantages: (1) MFAC has a simple structure, a low computational load, is straightforward to implement, and has strong robustness; (2) external test signals or a training process are not required for MFAC, as they are necessary for data-driven methods based on neural networks; (3) under certain practical assumptions, MFAC can provide the monotonic convergence of the tracking error of the closed-loop system as well as the stability of bounded input and bounded output, which is a critical property that differentiates it from other data-driven control approaches [13].

The neural network [16] is a network structure composed of numerous parallel computing artificial neurons connected by algorithms, and high computation performance can be achieved by connecting basic neurons according to a certain logic. In addition to approximating nonlinear functions, the neural network is also capable of being adaptable and self-learning. From the perspective of control, automatic control has been pursuing stability, rapidity, robustness and adaptability. The main advantages of applying neural networks to control are [17]: (1) a neural network can be regarded as a specific nonlinear function capable of approximating any nonlinear system in the absence of model information; (2) a neural network is calculated in parallel, with fast calculation speed and high fault tolerance; (3) A neural network can be used to simulate the dynamic models of unknown systems; and (4) the neural network can reduce calculation errors through online learning. Some theoretical results have been derived from research using a combination of DDC methods and neural networks. Radacm et al. [18] proposed a novel DDC control method that combines VRFT and AAC for linear ORM tracking, and the control learning scheme is model-free with respect to the process model. Liu et al. [19] developed an event-based data-driven model-free adaptive controller design algorithm, and an aperiodic neural network weight update law is introduced to estimate the controller parameters in this method; Hao et al. [20] developed a data-driven tracking control method by employing an improved PID neural network and Cohen–Coon approach for a class nonlinear time-varying systems, and the stability of the closed loop system based on the proposed method is proven via the Lyapunov stability theory. Rodrigo et al. [21] proposed an auto-tune PID-like controller with neural networks to help the underwater vehicle adaptively switch driving mode when encountering ocean currents, and experimental results show that the underwater vehicle can achieve a smaller position tracking error based on the proposed method. Sun et al. [22] introduced adaptive neural networks (NNs) for control design to suppress the vibrations of a flexible robotic manipulator. The system is modeled via the lumped spring-mass approach to improve the accuracy in describing the elastic deflection of the flexible manipulator.

Individual parameters are critical in FFMFAC, since they influence the stability and control performance of the controlled system. Normally, these parameters have predefined fixed values [15]. However, under actual working conditions, as the state of the controlled system changes, these parameters should be fine-adjusted to ensure control performance. However, completing the parameters adjustment is a labor-intensive and time-consuming work, and incorrect values might result in reduced control performance. Therefore, the online adjustment of these vital parameters is of great practical significance. Up to this point, only a few theoretical results on the adjustment of MFAC parameters have been published. Zhu et al. [23] proposed an enhanced MFAC method, which introduces the RBF neural network to adjust the controller parameters. The stability of the proposed method is guaranteed by rigorous theoretical analysis. Chen and Lu [24] introduced BP-based compact-form MFAC which can perform parameters by online adjustment. However, the authors did not apply the method to an actual test simulation for performance evaluation. Gao et al. [25] employed the PSO method to iteratively find the optimal parameters of MFAC to improve the control performance. However, the iterative calculation of optimal values consumes significant computing resources, which is unsuitable for practical control problems.

In the actual industrial production process, with the rapid increase in industrial data and the more complex controlled system, the difficulty of employing feedforward propagation neural networks to perform online parameter adjustment is increasing [26]. It has been demonstrated in prior study [27] that LSTMs [28] can adjust parameters online in the compact-form MFAC, and it has a stronger optimization influence on the compact-form MFAC than the BP neural network. In addition, considering the fact that FFMFAC has the most parameters to be adjusted among MFAC variants, the amount of calculation required to adjust these parameters is considerable. As a result, LSTMs are used to adjust FFMFAC parameters online.

Except in online parameter adjustment, changes in PG values will become complicated when the controlled system exhibits significant nonlinearity [29]. If only the projection method of MFAC is utilized to calculate PG values alone, the estimated values may significantly deviate from the ideal values, impairing the control performance. As previous research has found, the PG values of MFAC remain the initial constant values during part of the time interval in the three-tank system simulation [27]. This demonstrates that when dealing with control problems with significant time delays, the default PG estimation projection algorithm in MFAC has a certain probability of triggering the reset mechanism, resulting in the method failing to capture the nonlinear properties of the controlled system. Therefore, optimizing the PG estimation method has important research significance and practical application value. The FNN [30] has a strong function approximation ability as well as logical reasoning capability, and it can be employed to estimate FFMFAC’s PG values. Furthermore, the FNN’s topology is simple, ensuring its calculation efficiency.

Given the two challenges inherent in the ordinary FFMFAC method: (1) vital parameters in FFMFAC need to be sensitively and instantaneously adjusted in response to changes in the controlled system; (2) PG values should be estimated more accurately in the FFDL method of FFMFAC. An enhanced FFMFAC is proposed to achieve the desired control of discrete-time nonlinear systems. EFFMFAC is characterized by utilizing the measured I/O data of the controlled system for the online training of all introduced neural networks and does not involve offline training and specific models.

The significant contributions of this paper are as follows:

1. LSTMs are utilized to sensitively and instantaneously adjust vital parameters online. This employs several gates to process the data flow of the controlled system, and each gate is capable of capturing the dynamic characteristics of input data, alleviating the gradient problems in the RNN and improving the tracking performance of EFFMFAC.
2. FNNs are employed to estimate PG values in the FFDL method of FFMFAC, which is completely dependent on the controlled system’s measured I/O data. The FNN refers to a local approximation methodology with the inference ability of a fuzzy system, and its convergence speed is fast. Therefore, it is well suited for nonlinear calculations to achieve the accurate real-time estimation of PG values.
3. A complete enhanced control method is proposed to achieve the precise control of the SISO discrete-time nonlinear system, in which the parameters’ online adjustment module and PG estimation module work together to improve the control performance through online training. Scientific and thorough simulations were conducted to verify the rationality and superiority of EFFMFAC.

The following is the outline for this paper: Section 2 is dedicated to problem conceptualization. Section 3 describes the architecture and mathematical concepts of EFFMFAC, including the vital parameters’ online adjustment module and PG estimation module; Section 4 is the experimental part, in which EFFMFAC is shown to be superior and stable in all simulations; Section 5 brings this paper to a close and discusses future research plans.

## 2. Problem Definition

A class of SISO discrete-time nonlinear systems is defined as follows [3]:

(1) $$y\left(k+1\right)=f\left(y\left(k\right),\cdots ,y\left(k-n_{y}\right),u\left(k\right),\cdots ,u\left(k-n_{u}\right)\right)$$

where y(k)∈R, u(k)∈R represent the system’s output and input at time *k*; $n_{y}$ and $n_{u}$ are two positive integers; $f\left(\cdots \right):R^{n_{u}+n_{y}+2}\mapsto R$ denotes an unknown nonlinear function.

Define $H_{Ly,Lu}\left(k\right)\in R^{Ly+Lu}$ as a vector containing the control input signal in the input-related sliding time window [k−Lu+1,k] and all system output signals in the output-related sliding time window [k−Ly+1,k], namely:

(2) $$H_{Ly,Lu}\left(k\right)={\left[y\left(k\right),\cdots ,y\left(k-Ly+1\right),u\left(k\right),\cdots ,u\left(k-Lu+1\right)\right]}^{T}$$

where Ly ($0⩽Ly⩽n_{y}$) and Lu ($0⩽Lu⩽n_{u}$) are, respectively, the control output linearization length and the control input linearization length.

The following two assumptions are provided for the system (1) [3]:

**Assumption** **A1.** *Unknown nonlinear function f(⋯) has continuous partial derivatives with respect to each variable.*

**Assumption** **A2.** *The SISO discrete-time nonlinear system (1) satisfies the generalized Lipschitz condition: for any $k_{1}\neq k_{2},k_{1},k_{2}⩾0$ and $H_{Ly,Lu}\left(k_{1}\right)\neq H_{Ly,Lu}\left(k_{2}\right)$, then $\left|y\left(k_{1}+1\right)-y\left(k_{2}+1\right)\right|⩽b∥H_{Ly,Lu}\left(k_{1}\right)-H_{Ly,Lu}\left(k_{2}\right)∥$ is established, where b is a constant.*

Practically speaking, the assumptions made above related to the controlled system (1) are acceptable. Assumption 1 is a common constraint condition in control system design. Assumption 2 is a restriction on the upper bound of the system output change rate. From the energy perspective, the bounded input and output energy changes in the previous time should produce the bounded output energy changes at the current time. Numerous existing systems satisfy Assumption 2, such as liquid-level control systems and pressure control systems.

Define $\Delta H_{Ly,Lu}\left(k\right)=H_{Ly,Lu}\left(k\right)-H_{Ly,Lu}\left(k-1\right)$; the following theorem proposes a full-form dynamic linearization (FFDL) method for system (1):

**Theorem** **1.** *For system (1) that satisfies Assumption 1 and Assumption 2, given $0⩽Ly⩽n_{y}$ and $0⩽Lu⩽n_{u}$, when $∥\Delta H_{Ly,Lu}\left(k\right)∥\neq 0$, there exists a time-varying parameter vector $\phi_{f,Ly,Lu}\left(k\right)\in R^{Ly+Lu}$ named pseudo gradient (PG) that can transfer the SISO discrete-time nonlinear system (1) into the following FFDL model:*

(3) $$\Delta y\left(k+1\right)=\phi_{f,Ly,Lu}^{T}\left(k\right)\Delta H_{Ly,Lu}\left(k\right)$$

*and for any time k, $\phi_{f,L_{y},L_{u}}\left(k\right)={\left[\phi_{1}\left(k\right),\cdots ,\phi_{L_{y}}\left(k\right),\phi_{L_{y}+1}\left(k\right),\cdots ,\phi_{L_{y}+L_{u}}\left(k\right)\right]}^{T}$ is bounded.*

**Proof** **of** **Theorem** **1.** According to SISO discrete-time nonlinear system (1), Δy(k+1) can be calculated as follows:

(4) $$\begin{array}{c} \Delta y\left(k+1\right)=f\left(y\left(k\right),\cdots ,y\left(k-n_{y}\right),u\left(k\right),\cdots ,u\left(k-n_{u}\right)\right)\\ \begin{array}{c} \;-f\left(y\left(k-1\right),\cdots ,y\left(k-n_{y}-1\right),u\left(k-1\right),\cdots ,u\left(k-n_{u}-1\right)\right)\\ =f\left(y\left(k\right),\cdots ,y\left(k-L_{y}+1\right),y\left(k-L_{y}\right),\cdots ,y\left(k-n_{y}\right),\right.\\ \;\left.u\left(k\right),\cdots ,u\left(k-L_{u}+1\right),u\left(k-L_{u}\right),\cdots ,u\left(k-n_{u}\right)\right)\\ -f\left(y\left(k-1\right),\cdots ,y\left(k-L_{y}\right),y\left(k-L_{y}\right),\cdots ,y\left(k-n_{y}\right),\right.\\ \;\left.u\left(k-1\right),\cdots ,u\left(k-L_{u}\right),u\left(k-L_{u}\right),\cdots ,u\left(k-n_{u}\right)\right)\\ +f\left(y\left(k-1\right),\cdots ,y\left(k-L_{y}\right),y\left(k-L_{y}\right),\cdots ,y\left(k-n_{y}\right),\right.\\ \;\left.u\left(k-1\right),\cdots ,u\left(k-L_{u}\right),u\left(k-L_{u}\right),\cdots ,u\left(k-n_{u}\right)\right)\\ -f\left(y\left(k-1\right),\cdots ,y\left(k-L_{y}\right),y\left(k-L_{y}-1\right),\cdots ,y\left(k-n_{y}-1\right),\right.\\ \;\left.u\left(k-1\right),\cdots ,u\left(k-L_{u}\right),u\left(k-L_{u}-1\right),\cdots ,u\left(k-n_{u}-1\right)\right) \end{array} \end{array}$$

Define variable ψ(k) as follows:

(5) $$\begin{array}{cc} \psi (k)≜f & \left(y\left(k-1\right),\cdots ,y\left(k-L_{y}\right),y\left(k-L_{y}\right),\cdots ,y\left(k-n_{y}\right),\right.\\ \; & \left.u\left(k-1\right),\cdots ,u\left(k-L_{u}\right),u\left(k-L_{u}\right),\cdots ,u\left(k-n_{u}\right)\right)\\ - & f\left(y\left(k-1\right),\cdots ,y\left(k-L_{y}\right),y\left(k-L_{y}-1\right),\cdots ,y\left(k-n_{y}-1\right),\right.\\ \; & \left.u\left(k-1\right),\cdots ,u\left(k-L_{u}\right),u\left(k-L_{u}-1\right),\cdots ,u\left(k-n_{u}-1\right)\right) \end{array}$$

Equation (4) can be expressed as follows using Assumption 1 and the Cauchy Mean Value Theorem:

(6) $$\begin{array}{cc} \Delta y(k+1)= & \frac{\partial f^{*}}{\partial y(k)}\Delta y\left(k\right)+\cdots +\frac{\partial f^{*}}{\partial y\left(k-L_{y}\right)}\Delta y\left(k-L_{y}+1\right)\\ \; & +\frac{\partial f^{*}}{\partial u(k)}\Delta u\left(k\right)+\cdots +\frac{\partial f^{*}}{\partial u\left(k-L_{u}\right)}\Delta u\left(k-L_{u}+1\right)+\psi \left(k\right) \end{array}$$

where $\partial f^{*}/\partial y\left(k-i\right),0⩽i⩽L_{y}-1$ and $\partial f^{*}/\partial u\left(k-j\right),0⩽j⩽L_{u}-1$, respectively, represent the partial derivative of f(⋯) with respect to the (i+1)th variable and the partial derivative of f(⋯) with respect to the $\left(n_{y}+2+j\right)th$ variable at a point between:

(7) $$\begin{array}{c} \left[y\left(k\right),\cdots ,y\left(k-L_{y}+1\right),y\left(k-L_{y}\right),\cdots ,y\left(k-n_{y}\right),\right.\\ {\left.u\left(k\right),\cdots ,u\left(k-L_{u}+1\right),u\left(k-L_{u}\right),\cdots ,u\left(k-n_{u}\right)\right]}^{T} \end{array}$$

and:

(8) $$\begin{array}{c} \left[y\left(k-1\right),\cdots ,y\left(k-L_{y}\right),y\left(k-L_{y}\right),\cdots ,y\left(k-n_{y}\right),\right.\\ {\left.u\left(k-1\right),\cdots ,u\left(k-L_{u}\right),u\left(k-L_{u}\right),\cdots ,u\left(k-n_{u}\right)\right]}^{T} \end{array}$$

The following data equation with variable η(k) is considered:

(9) $$\begin{array}{cc} \psi (k) & =\eta^{T}\left(k\right){\left[\Delta y\left(k\right),\cdots ,\Delta y\left(k-L_{y}+1\right),\Delta u\left(k\right),\cdots ,\Delta u\left(k-L_{u}+1\right)\right]}^{T}\\ \; & =\eta^{T}\left(k\right)\Delta H_{L_{v},L_{u}}\left(k\right) \end{array}$$

since $∥\Delta H_{L_{y},L_{u}}\left(k\right)∥\neq 0$ is not equal to 0, Equation (9) has at least one solution $\eta^{*}\left(k\right)$ and the variable $\phi_{f,L_{y},L_{u}}\left(k\right)$ is defined as follows:

(10) $$\phi_{f,L_{y},L_{u}}\left(k\right)=\eta^{*}\left(k\right)+{\left[\frac{\partial f^{*}}{\partial y(k)},\cdots ,\frac{\partial f^{*}}{\partial y\left(k-L_{y}\right)},\frac{\partial f^{*}}{\partial u(k)},\cdots ,\frac{\partial f^{*}}{\partial u\left(k-L_{u}\right)}\right]}^{T}$$

then Equation (6) can be written as the FFDL model of Equation (3). This completes the proof. □

The FFDL model (3) plays the role of an equivalent dynamic linear representation of the SISO discrete-time nonlinear system (1), which has a simple incremental form that fundamentally differs from the traditional models. When designing a control scheme for a discrete-time nonlinear system, there are two main criteria functions: the one-step forward prediction error criterion function and the weighted one-step forward prediction error criterion function. The former is prone to producing an excessively large control input signal when the error fluctuates significantly, which will affect the identification of characteristic parameters and cause output oscillations. The latter may reduce the tracking performance of the controller and produce steady-state tracking errors [31]. In order to overcome the shortcomings of the above two criterion functions, the following criterion function is considered [3]:

(11) $$J\left(u\left(k\right)\right)={\left|y^{*}\left(k+1\right)-y\left(k+1\right)\right|}^{2}+\lambda {\left|u\left(k\right)-u\left(k-1\right)\right|}^{2}$$

where $y^{*}\left(k+1\right)$ is the desired output signal, and λ>0 is a weighting factor that restricts the change in the control input and is commonly used in control system design since it ensures that the control input signal is smooth. The criterion function (11) contains two parts, the first term ${\left|y^{*}\left(k+1\right)-y\left(k+1\right)\right|}^{2}$ is provided to massively reduce system error, while the second term ${\lambda |u\left(k\right)-u\left(k-1\right)|}^{2}$ is provided to avoid excessive control input changes and eliminate steady-state tracking errors. These two terms broaden the application of criterion function (11) to nonlinear control problems. The optimal solution may be obtained by substituting the FFDL model (3) into the criterion function (11), taking the derivative of u(k), and setting it equal to zero.

**Figure 1:**
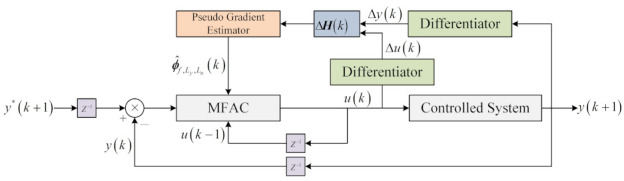
Diagram of FFMFAC.

The diagram of FFMFAC is illustrated in Figure 1, with regard to the system (1), and the specific control scheme of FFMFAC is expressed as follows:

(12) $$\begin{array}{cc} {\hat{\phi }}_{f,Ly,Lu}\left(k\right) & ={\hat{\phi }}_{f,Ly,Lu}\left(k-1\right)\\ \; & +\frac{\eta \Delta H_{Ly,Lu}\left(k-1\right)\left(y\left(k\right)-y\left(k-1\right)-{\hat{\phi }}_{f,Ly,Lu}^{T}\left(k-1\right)\Delta H_{Ly,Lu}\left(k-1\right)\right)}{\mu +{∥\Delta H_{Ly,Lu}\left(k-1\right)∥}^{2}} \end{array}$$

(13) $$\begin{array}{c} {\hat{\phi }}_{f,Ly,Lu}\left(k\right)={\hat{\phi }}_{f,Ly,Lu}\left(1\right)\;\\ if\left|{\hat{\phi }}_{f,Ly,Lu}\left(k\right)\right|\leq \varepsilon \;or∥\Delta H_{Ly,L_{u}}\left(k-1\right)∥⩽\varepsilon \;or\;sign\left({\hat{\phi }}_{Ly+1}\left(k\right)\right)\neq sign\left({\hat{\phi }}_{Ly+1}\left(1\right)\right) \end{array}$$

(14) $$\begin{array}{cc} u(k)= & u\left(k-1\right)+\frac{\rho_{Ly+1}{\hat{\phi }}_{Ly+1}\left(k\right)\left(y^{*}\left(k+1\right)-y\left(k\right)\right)}{\lambda +{\left|{\hat{\phi }}_{Ly+1}\left(k\right)\right|}^{2}}\\ \; & -\frac{{\hat{\phi }}_{Ly+1}\left(k\right)\sum_{i=1}^{Ly}\rho_{i}{\hat{\phi }}_{i}\left(k\right)\Delta y\left(k-i+1\right)}{\lambda +{\left|{\hat{\phi }}_{Ly+1}\left(k\right)\right|}^{2}}\\ \; & -\frac{{\hat{\phi }}_{Ly+1}\left(k\right)\sum_{i=Ly+2}^{Ly+Lu}\lambda {\hat{\phi }}_{i}\left(k\right)\Delta u\left(k-Ly-i+1\right)}{\lambda +{\left|{\hat{\phi }}_{Ly+1}\left(k\right)\right|}^{2}} \end{array}$$

where η∈(0,2], μ>0, and $\rho_{i}\in \left(0,1\right],i=1,2,\cdots ,Ly+Lu$ is the step factor. ${\hat{\phi }}_{f,Ly,Lu}\left(1\right)$ is the initial value of ${\hat{\phi }}_{f,Ly,Lu}\left(k\right)$. The PG reset mechanism (13) is used to improve the ability of the PG estimation method (12) to track time-varying parameters.

Unlike traditional model-based control methods, FFMFAC completes the controller design by utilizing the controlled system’s online input and output data and has nothing to do with the controlled system’s dynamic model. Since ${\hat{\phi }}_{f,Ly,Lu}$ is insensitive to time-varying parameters, FFMFAC exhibits strong adaptability and robustness. In addition, compared with the CFDL method and PFDL method, the FFDL method also considers the influence of the historical I/O changes of the controlled system on the current output changes to better reflect the dynamic characteristics of the controlled system. Due to the introduction of more penalty factors $\rho_{1},\rho_{2},\cdots ,\rho_{Ly+Lu}$, FFMFAC has stronger design flexibility and applicability [3].

Parameters $\rho_{i}$ and λ have been shown to be significantly important in the design of FFMFAC by several studies [24,25,27].These studies emphasize the significance of fine-adjusting these parameters in response to changes in the controlled system, with theoretical analysis and simulation findings indicating how improper parameter selection can impact the stability of the controller, resulting in reduced control performance. Furthermore, it should be stressed that PG values should be precisely estimated in order to realize the FFMFAC. Apart from that, since PG values are time-varying and the mathematical model of the controlled system is unavailable, it is a challenge to calculate the precise values of PG. As a consequence, it is vital to optimize the PG estimate method of FFMFAC to calculate more accurate calculation values. Motivated by the above, an enhanced FFMFAC design is proposed to address the aforementioned issues.

## 3. The Proposed Enhanced FFMFAC Method

Motivated by the above challenges, a neural network-based enhanced FFMFAC was proposed to sensitively adjust the vital parameters online and accurately estimate PG values. To be more precise, EFFMFAC introduces LSTMs to complete the parameter adjustment of λ and $\rho_{1},\rho_{2},\cdots ,\rho_{Ly+Lu}$ online, and also uses FNNs to realize the PG values estimation of FFMFAC. All of the deployed neural networks are trained online based on measured data to improve the control performance of EFFMFAC.

### 3.1. LSTM-Based Parameters Online Adjustment Module

**Figure 2:**
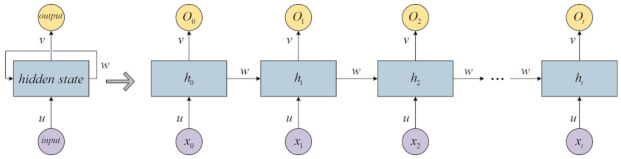
Structure of the RNN.

**Figure 3:**
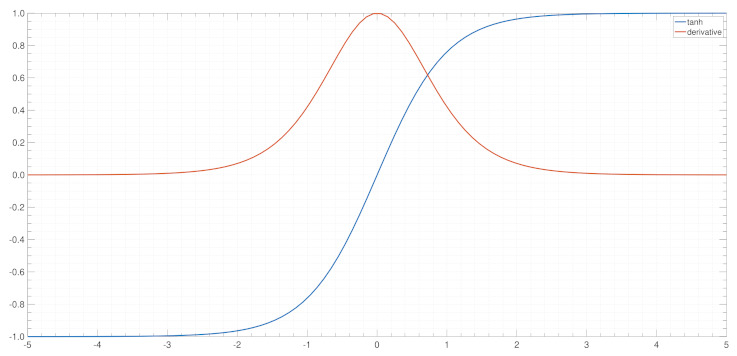
Curves of the tanh activation function and its derivative.

Jordan [32] first proposed the recurrent neural network in 1986, which can describe dynamic time behavior. As illustrated in Figure 2, unlike feedforward neural networks that accept inputs with a more specific structure, RNN cyclically transmits the hidden states in its own network, so it can accept a wider range of time series inputs:

The forward propagation calculation of the RNN is expressed as below:

(15) $$h_{t}=\tanh\left(ux_{t}+wh_{t-1}\right)$$

(16) $$O^{t}=g\left(vh_{t}\right)$$

where $x_{t}$, $h_{t}$ and $O_{t}$, respectively, represent the input, hidden state and output of RNN at time *t*, *u* presents the weight matrix of the input layer to the hidden layer, *v* presents the weight matrix of the hidden layer to the output layer and *w* presents the weight matrix of the hidden state at time t−1. tanh() is the activation function, and g(x) is the softmax activation function. However, RNN has the problem of gradient explosion or gradient vanishing [33] when backpropagating, which affects its wide application in actual scenes. As an example, consider the weight matrix *u* to be updated, and the partial derivative formula of *u* at time *t* is shown as follows:

(17) $$\begin{array}{cc} \frac{\partial L_{t}}{\partial u}= & \sum_{k=0}^{t}\frac{\partial L_{t}}{\partial O_{t}}\frac{\partial O_{t}}{\partial h_{t}}\left(\prod_{j=k+1}^{t}\frac{\partial h_{j}}{\partial h_{j-1}}\right)\frac{\partial h_{k}}{\partial u}\\ = & \sum_{k=0}^{t}\frac{\partial L_{t}}{\partial O_{t}}\frac{\partial O_{t}}{\partial h_{t}}\left(\prod_{j=k+1}^{t}\tanh^{\prime }w\right)\frac{\partial h_{k}}{\partial u} \end{array}$$

where $L_{t}$ is the loss function. As illustrated in Figure 3, it can be found that the value of $\tanh^{\prime }$ is less than 1. When the coefficient *w* value is between 0 and 1, the value of the term $\prod_{j=k+1}^{t}\tanh^{\prime }w$ will gradually decrease as time *t* increases until it reaches zero. Conversely, if coefficient *w* is very large and $\tanh^{\prime }w$ is greater than 0, the value of term $\prod_{j=k+1}^{t}\tanh^{\prime }w$ will tend to infinity as time increases. The above two cases are defined as the gradient vanishing and gradient exploding in RNN, which limit its practical widespread application:

Hochreiter and Schmidhuber proposed the LSTM [34] in 1997. In contrast to RNN, it can alleviate the gradient problems with the gate mechanism [35]. The core cause of the RNN gradient problems is the term $\partial h_{t}$/$\partial h_{t-1}$ in Equation (17), and the similar term ∂c(k)/∂c(k−1) in the LSTM backpropagation calculation is expanded as below:

(18) $$c\left(k\right)=f\left(k\right)⊙c\left(k-1\right)+i\left(k\right)⊙\tilde{c}\left(k\right)$$

(19) $$\begin{array}{cc} \frac{\partial c(k)}{\partial c(k-1)} & =\frac{\partial c(k)}{\partial f(k)}\frac{\partial f(k)}{\partial h(k-1)}\frac{\partial h(k-1)}{\partial c(k-1)}+\frac{\partial c(k)}{\partial i(k)}\frac{\partial i(k)}{\partial h(k-1)}\frac{\partial h(k-1)}{\partial c(k-1)}\\ \; & +\frac{\partial c(k)}{\partial \tilde{c}\left(k\right)}\frac{\partial \tilde{c}\left(k\right)}{\partial h(k-1)}\frac{\partial h(k-1)}{\partial c(k-1)}+\frac{\partial c(k)}{\partial c(k-1)} \end{array}$$

where c(k) and $\tilde{c}\left(k\right)$ are the cell state and the candidate cell state, respectively, f(k) and i(k) represent the input gate and the forget gate, respectively. Partial derivatives ∂c(k)/∂c(k−1) in Equation (19) can be calculated as below:

(20) $$\begin{array}{cc} \frac{\partial c(k)}{\partial c(k-1)} & =c\left(k-1\right)\sigma^{\prime }\left(\cdot \right)w_{f}*o\left(k-1\right)\tanh^{\prime }\left(c(k-1)\right)\\ \; & +\tilde{c}\left(k\right)\sigma^{\prime }\left(\cdot \right)w_{i}*o\left(k-1\right)\tanh^{\prime }\left(c(k-1)\right)\\ \; & +i\left(k\right)\tanh^{\prime }\left(\cdot \right)w_{c}*o\left(k-1\right)\tanh^{\prime }\left(c(k-1)\right)+f\left(k\right) \end{array}$$

where $w_{f}$, $w_{f}$ and $w_{c}$ are the weight coefficients and σ is the sigmoid activation function. In contrast to Equation (17), ∂c(k)/∂c(k−1) is a polynomial including forget gate f(k)∈[0,1], whose value range at any time may be distributed between 0 and 1 or greater than 1. As time step *t* increases, it is not guaranteed that ∂c(k)/∂c(k−1) will converge to zero or infinity, which can avoid gradient vanishing and gradient exploding in RNN. Therefore, LSTMs are introduced to complete the parameters adjustment work of λ and $\rho_{1},\rho_{2},\cdots ,\rho_{Ly+Lu}$ online, and the architecture of the parameters online adjustment module based on LSTMs is shown in Figure 4.

The input to this module contains the system error information as well as gradient information concerning the parameters to be adjusted, which are expressed below:

(21) $$\begin{array}{cc} x_{error}= & \left[e\left(k\right),e\left(k\right)-e\left(k-1\right),\sum_{t=0}^{k}e\left(k\right)\right]\\ x_{u_{\lambda }}= & \left[\frac{\partial u(k-1)}{\partial \lambda },\frac{\partial u(k-2)}{\partial \lambda },\frac{\partial u(k-3)}{\partial \lambda }\right]\\ x_{u_{\rho }}= & \left[\frac{\partial u(k-1)}{\partial \rho_{1}},\frac{\partial u(k-2)}{\partial \rho_{1}},\frac{\partial u(k-3)}{\partial \rho_{1}},\cdots \right.\\ \; & \frac{\partial u(k-1)}{\partial \rho_{l}},\frac{\partial u(k-2)}{\partial \rho_{l}},\frac{\partial u(k-3)}{\partial \rho_{l}},\cdots \\ \; & \left.\frac{\partial u(k-1)}{\partial \rho_{Ly+Lu}},\frac{\partial u(k-2)}{\partial \rho_{Ly+Lu}},\frac{\partial u(k-3)}{\partial \rho_{Ly+Lu}}\right] \end{array}$$

where $x_{error}$ is the system error set, $x_{u_{\lambda }}$ and $x_{u_{\rho }}$ represent the gradient information sets. The input fed to LSTMs is denoted below:

(22) $$X\left(k\right)=\left[x_{error},x_{u_{\lambda }},x_{u_{\rho }}\right]$$

LSTMs perform forward propagation calculation, and all calculation formulas are expressed as follows:

(23) $$\begin{array}{cc} net_{fi}\left(k\right) & =w_{fi}\left[X(k),h(k-1)\right]+b_{fi}\\ f_{i}\left(k\right) & =sigmoid\left(net_{fi}\left(k\right)\right) \end{array}$$

(24) $$\begin{array}{cc} net_{Ii}\left(k\right) & =w_{Ii}\left[X(k),h(k-1)\right]+b_{Ii}\\ I_{i}\left(k\right) & =sigmoid\left(net_{Ii}\left(k\right)\right) \end{array}$$

(25) $$\begin{array}{cc} net_{\tilde{c}i}\left(k\right) & =w_{ci}\left[X(k),h(k-1)\right]+b_{ci}\\ {\tilde{c}}_{i}\left(k\right) & =tanh\left(net_{\bar{c}i}\left(k\right)\right) \end{array}$$

(26) $$c_{i}\left(k\right)=c_{i}\left(k-1\right)⊙f_{i}\left(k\right)+I_{i}\left(k\right)⊙{\tilde{c}}_{i}\left(k\right)$$

(27) $$\begin{array}{cc} net_{oi}\left(k\right) & =w_{\mathrm{oi}}\left[X(k),h(k-1)\right]+b_{\mathrm{oi}}\\ o_{i}\left(k\right) & =sigmoid\left(net_{oi}\left(k\right)\right) \end{array}$$

(28) $$h_{i}\left(k\right)=o_{i}\left(k\right)⊙tanh\left(c_{i}\left(k\right)\right),i=1,2,\ldots hidnum$$

(29) $$onet_{l}\left(k\right)=w_{mh}h_{i}\left(k\right)+b_{mh}$$

(30) $$out_{l}\left(k\right)=\sigma \left(onet_{l}\left(k\right)\right)$$

where $out_{l}\left(k\right)$ is the output of the output layer, $f_{i}\left(k\right)$ and $o_{i}\left(k\right)$ are the output of the forget gate and output gate, respectively, $I_{i}\left(k\right)$ and ${\tilde{c}}_{i}\left(k\right)$ are the components of the input gate output, $h_{i}\left(k\right)$ is the hidden layer output, $w_{fi}$, $w_{li}$, $w_{ci}$, $w_{oi}$ and $w_{mh}$ are the weight coefficients; $b_{fi}$, $b_{li}$, $b_{ci}$, $b_{oi}$ and $b_{mh}$ are the bias coefficients, hidnum is the number of hidden layers. sigmoid and tanh are both activation functions, and their formulas are expressed as follows:

(31) $$sigmoid\left(z\right)=\frac{1}{1+e^{-z}}$$

(32) $$tanh\left(z\right)=\frac{e^{z}-e^{-z}}{e^{z}+e^{-z}}$$

The particular values of all parameters to be adjusted can be determined according to Equation (30):

(33) $$\begin{array}{c} \lambda =out_{l1}\left(k\right)\\ \rho_{l}=out_{l(l+1)}\left(k\right),l=1,2,\cdots ,Ly+Lu \end{array}$$

The control input u(k) can be calculated with the systematic error e(k). Take the one-step-ahead squared error as the indicator function:

(34) $$J=\frac{1}{2}e{\left(k+1\right)}^{2}=\frac{1}{2}{\left(y^{*}\left(k+1\right)-y\left(k+1\right)\right)}^{2}$$

Weight and bias coefficients are updated by utilizing the chain-based backpropagation algorithm (BPTT). Only the update calculation of the weight coefficients are given for brevity’s sake:

(35) $$\begin{array}{cc} w_{fi}\left(k+1\right)= & w_{fi}\left(k\right)-\eta \frac{\partial J}{\partial w_{fi}}\\ \frac{\partial J}{\partial w_{fi}}= & \frac{\partial J}{\partial y(k+1)}\frac{\partial y(k+1)}{\partial u(k)}\frac{\partial u(k)}{\partial out_{l}\left(k\right)}\frac{\partial out_{l}\left(k\right)}{\partial onet_{l}\left(k\right)}\frac{\partial onet_{l}\left(k\right)}{\partial h_{i}\left(k\right)}\frac{\partial h_{i}\left(k\right)}{\partial c_{i}\left(k\right)}\frac{\partial c_{i}\left(k\right)}{\partial f_{i}\left(k\right)}\frac{\partial f_{i}\left(k\right)}{\partial net_{fi}\left(k\right)}\frac{\partial net_{fi}\left(k\right)}{\partial w_{fi}} \end{array}$$

(36) $$\begin{array}{cc} w_{Ii}\left(k+1\right)= & w_{Ii}\left(k\right)-\eta \frac{\partial J}{\partial w_{Ii}}\\ \frac{\partial J}{\partial w_{Ii}}= & \frac{\partial J}{\partial y(k+1)}\frac{\partial y(k+1)}{\partial u(k)}\frac{\partial u(k)}{\partial out_{l}\left(k\right)}\frac{\partial out_{l}\left(k\right)}{\partial onet_{l}\left(k\right)}\frac{\partial onet_{l}\left(k\right)}{\partial h_{i}\left(k\right)}\frac{\partial h_{i}\left(k\right)}{\partial c_{i}\left(k\right)}\frac{\partial c_{i}\left(k\right)}{\partial I_{i}\left(k\right)}\frac{\partial I_{i}\left(k\right)}{\partial net_{Ii}\left(k\right)}\frac{\partial net_{Ii}\left(k\right)}{\partial w_{Ii}} \end{array}$$

(37) $$\begin{array}{cc} w_{ci}\left(k+1\right)= & w_{ci}\left(k\right)-\eta \frac{\partial J}{\partial w_{ci}}\\ \frac{\partial J}{\partial w_{ci}}= & \frac{\partial J}{\partial y(k+1)}\frac{\partial y(k+1)}{\partial u(k)}\frac{\partial u(k)}{\partial out_{l}\left(k\right)}\frac{\partial out_{l}\left(k\right)}{\partial onet_{l}\left(k\right)}\frac{\partial onet_{l}\left(k\right)}{\partial h_{i}\left(k\right)}\frac{\partial h_{i}\left(k\right)}{\partial c_{i}\left(k\right)}\frac{\partial c_{i}\left(k\right)}{\partial {\tilde{c}}_{i}\left(k\right)}\frac{\partial {\tilde{c}}_{i}\left(k\right)}{\partial net_{ci}\left(k\right)}\frac{\partial net_{\bar{c}i}\left(k\right)}{\partial w_{ci}} \end{array}$$

(38) $$\begin{array}{cc} w_{oi}\left(k+1\right)= & w_{oi}\left(k\right)-\eta \frac{\partial J}{\partial w_{oi}}\\ \frac{\partial J}{\partial w_{oi}}= & \frac{\partial J}{\partial y(k+1)}\frac{\partial y(k+1)}{\partial u(k)}\frac{\partial u(k)}{\partial out_{l}\left(k\right)}\frac{\partial out_{l}\left(k\right)}{\partial onet_{l}\left(k\right)}\frac{\partial onet_{l}\left(k\right)}{\partial h_{i}\left(k\right)}\frac{\partial h_{i}\left(k\right)}{\partial o_{i}\left(k\right)}\frac{\partial o_{i}\left(k\right)}{\partial net_{oi}\left(k\right)}\frac{\partial net_{oi}\left(k\right)}{\partial w_{oi}} \end{array}$$

(39) $$\begin{array}{cc} w_{mh}\left(k+1\right)= & w_{mh}\left(k\right)-\eta \frac{\partial J}{\partial w_{mh}}\\ \frac{\partial J}{\partial w_{mh}}= & \frac{\partial J}{\partial y(k+1)}\frac{\partial y(k+1)}{\partial u(k)}\frac{\partial u(k)}{\partial out_{l}\left(k\right)}\frac{\partial out_{l}\left(k\right)}{\partial onet_{l}\left(k\right)}\frac{\partial onet_{l}\left(k\right)}{\partial w_{mh}}\;\;\;\;\;\;\;\;\;\; \end{array}$$

where η represents the learning rate. The update process of bias coefficients is similar to that of the weight coefficients. The paramount term in the weight coefficients update calculation is $\partial u\left(k\right)/\partial out_{l}\left(k\right)$, which is the partial derivative of u(k) with respect to vital parameters λ and $\rho_{l}\left(l=1,\cdots ,Ly+Lu\right)$, and the formulas are expressed as below:

(40) $$\begin{array}{cc} \frac{\partial u(k)}{\partial \lambda }= & -\frac{\rho_{Ly+1}{\hat{\phi }}_{L_{y}+1}\left(k\right)\left(y^{*}\left(k+1\right)-y\left(k\right)\right)}{{\left(\lambda +{\left|{\hat{\phi }}_{Ly+1}\left(k\right)\right|}^{2}\right)}^{2}}\;\;\;\;\;\;\;\\ \; & +\frac{{\hat{\phi }}_{Ly+1}\left(k\right)\sum_{i=1}^{Ly}\rho_{i}{\hat{\phi }}_{i}\left(k\right)\Delta y\left(k-i+1\right)}{{\left(\lambda +{\left|{\hat{\phi }}_{Ly+1}\left(k\right)\right|}^{2}\right)}^{2}}\\ \; & +\frac{{\hat{\phi }}_{Ly+1}\left(k\right)\sum_{i=Ly+2}^{Ly+Lu}\rho_{i}{\hat{\phi }}_{i}\left(k\right)\Delta u\left(k-Ly-i+1\right)}{{\left(\lambda +{\left|{\hat{\phi }}_{Ly+1}\left(k\right)\right|}^{2}\right)}^{2}} \end{array}$$

(41) $$\frac{\partial u(k)}{\partial \rho_{l}}=\left\{\begin{array}{cc} \; & -\frac{{\hat{\phi }}_{Ly+1}\left(k\right)\sum_{i=1}^{Ly}{\hat{\phi }}_{i}\left(k\right)\Delta y\left(k-i+1\right)}{\left(\lambda +{\left|{\hat{\phi }}_{Ly+1}\left(k\right)\right|}^{2}\right)},l=1\leq l\leq Ly\\ \; & \frac{{\hat{\phi }}_{L_{y}+1}\left(k\right)\left(y^{*}\left(k+1\right)-y\left(k\right)\right)}{\left(\lambda +{\left|{\hat{\phi }}_{Ly+1}\left(k\right)\right|}^{2}\right)},l=Ly+1\\ \; & -\frac{{\hat{\phi }}_{Ly+1}\left(k\right)\sum_{i=Ly+2}^{Ly+Lu}{\hat{\phi }}_{i}\left(k\right)\Delta u\left(k-Ly-i+1\right)}{\left(\lambda +{\left|{\hat{\phi }}_{Ly+1}\left(k\right)\right|}^{2}\right)},Ly+2\leq l\leq Ly+Lu \end{array}\right.$$

### 3.2. PG Estimation Based on FNNs

An FNN [30] is a form of hybrid intelligence algorithm; it is a multi-layer forward network that takes the complementarity of neural networks and fuzzy systems into account. In the structure of FNN, the input and output nodes are used to represent the I/O signals of the fuzzy system, and the hidden layer nodes are used to represent the membership function and fuzzy rules. The parallel processing capability greatly improves the inference ability of the fuzzy system. In addition, the FNN has adaptive learning and nonlinear representation capabilities [36]. Therefore, FNNs are utilized to estimate PG values in FFMFAC.

The ’if−then’ fuzzy inference rule of the FNN is presented as follows [37]:

(42) $$\;\mathrm{If}\;x_{i}\;\mathrm{is}\;A_{1}^{i},x_{2}\;\mathrm{is}\;A_{2}^{i},\cdots ,x_{k}\;\mathrm{is}\;A_{k}^{i}\;\mathrm{then}\;y_{i}=p_{0}^{i}+p_{1}^{i}x_{1}+\cdots +p_{k}^{i}x_{k}$$

where $A_{j}^{i}$ is the fuzzy set of the fuzzy system, $p_{j}^{i}$ is the fuzzy system parameter and $y_{i}$ represents the output obtained according to the fuzzy rule. The input part (the if part) is fuzzy, and the output part (the then part) is certain. This fuzzy inference rule indicates that the output is a linear combination of the inputs.

**Figure 5:**
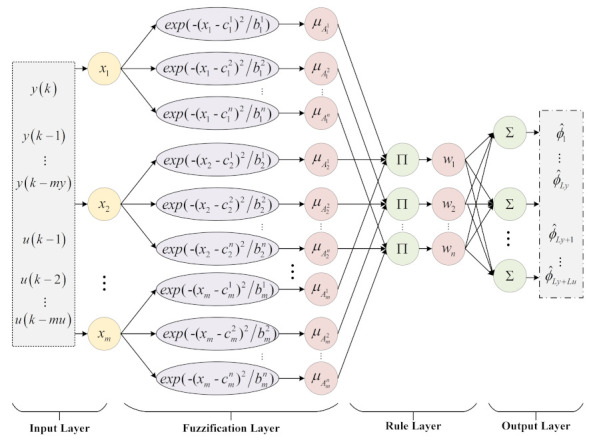
Structure of the PG estimation module.

The topology of the PG estimation module based on FNNs is shown in Figure 5. The input vector contains the system’s I/O information:

(43) x(k)=[y(k),…,y(k−my),u(k−1),…,u(k−mu)]

where my and mu are two integers. The membership of each input variable $x_{j}$ is calculated as follows:

(44) $$\mu_{A_{j}^{i}}=\exp\left(-{\left(x_{j}-c_{j}^{i}\right)}^{2}/b_{j}^{i}\right),j=1,2,\cdots ,num;i=1,2,\cdots ,n$$

where num is the number of variables in x(k), *n* is the number of fuzzy subsets, $c_{j}^{i}$ denotes the center of membership function and $b_{n}$ is the radius of membership function. Take the fuzzy calculation of each membership as shown below:

(45) $$\omega^{i}=\mu_{A_{j}^{1}}\left(x_{1}\right)*\mu_{A_{j}^{2}}\left(x_{2}\right)*\cdots *\mu_{A_{j}^{num}}\left(x_{num}\right)\;i=1,2,\cdots ,n$$

Combined with the output part of the fuzzy inference rule (42), the estimated PG value is calculated as follows:

(46) $$\hat{\phi }\left(k\right)=\sum_{i=1}^{n}\omega^{i}\left(p_{0}^{i}+p_{1}^{i}x_{1}+\cdots +p_{num}^{i}x_{num}\right)/\sum_{i=1}^{n}\omega^{i}$$

FNNs can output multiple estimated values when the number of output layers is set to Ly+Lu and the estimated PG values at time *k* are:

(47) $${\hat{\phi }}_{f,Ly,Lu}\left(k\right)=\left[{\hat{\phi }}_{1}\left(k\right),\cdots ,{\hat{\phi }}_{Ly}\left(k\right),\cdots ,{\hat{\phi }}_{Ly+Lu}\left(k\right)\right]$$

Take Equation (34) as the indicator function, learnable parameters in FNNs are updated as follows:

(48) $$\begin{array}{cc} p_{j}^{i}\left(k+1\right)= & p_{j}^{i}\left(k\right)-\beta \frac{\partial J}{\partial p_{j}^{i}\left(k\right)}+\alpha \Delta p_{j}^{i}\left(k\right)\\ \frac{\partial J}{\partial p_{j}^{i}\left(k\right)}= & \frac{\partial J}{\partial e(k+1)}\frac{\partial e(k+1)}{\partial y(k+1)}\frac{\partial y(k+1)}{\partial u(k)}\frac{\partial u(k)}{\partial \hat{\phi_{l}}\left(k\right)}\frac{\partial \hat{\phi_{l}}\left(k\right)}{\partial p_{j}^{i}\left(k\right)} \end{array}$$

(49) $$\begin{array}{cc} c_{j}^{i}\left(k+1\right)= & c_{j}^{i}\left(k\right)-\beta \frac{\partial J}{\partial c_{j}^{i}\left(k\right)}+\alpha \Delta c_{j}^{i}\left(k\right)\\ \frac{\partial J}{\partial c_{j}^{i}\left(k\right)}= & \frac{\partial J}{\partial e(k+1)}\frac{\partial e(k+1)}{\partial y(k+1)}\frac{\partial y(k+1)}{\partial u(k)}\frac{\partial u(k)}{\partial \hat{\phi_{l}}\left(k\right)}\frac{\partial \hat{\phi_{l}}\left(k\right)}{\partial \omega^{i}\left(k\right)}\frac{\partial \omega^{i}\left(k\right)}{\partial \mu_{A_{j}^{i}}\left(k\right)}\frac{\partial \mu_{A_{j}^{i}}\left(k\right)}{\partial c_{j}^{i}\left(k\right)} \end{array}$$

(50) $$\begin{array}{cc} b_{j}^{i}\left(k+1\right)= & b_{j}^{i}\left(k\right)-\beta \frac{\partial J}{\partial b_{j}^{i}\left(k\right)}+\alpha \Delta b_{j}^{i}\left(k\right)\\ \frac{\partial J}{\partial b_{j}^{i}\left(k\right)}= & \frac{\partial J}{\partial e(k+1)}\frac{\partial e(k+1)}{\partial y(k+1)}\frac{\partial y(k+1)}{\partial u(k)}\frac{\partial u(k)}{\partial \hat{\phi_{l}}\left(k\right)}\frac{\partial \hat{\phi_{l}}\left(k\right)}{\partial \omega^{i}\left(k\right)}\frac{\partial \omega^{i}\left(k\right)}{\partial \mu_{A_{j}^{i}}\left(k\right)}\frac{\partial \mu_{A_{j}^{i}}\left(k\right)}{\partial b_{j}^{i}\left(k\right)} \end{array}$$

where β and α are denoted as the learning rate and inertia coefficient, respectively. The partial derivatives of u(k) with respect to $\hat{\phi_{l}}\left(k\right)$ are expressed as below:

(51) $$\frac{\partial u(k)}{\partial \hat{\phi_{l}}\left(k\right)}=\left\{\begin{array}{cc} \; & -\frac{{\hat{\phi }}_{Ly+1}\left(k\right)\sum_{i=1}^{Ly}\rho_{i}\left(k\right)\Delta y\left(k-i+1\right)}{\left(\lambda +{\left|{\hat{\phi }}_{Ly+1}\left(k\right)\right|}^{2}\right)},l=1\leq l\leq Ly\\ \; & \frac{\left(\lambda -{\hat{\phi }}_{Ly+1}{\left(k\right)}^{2}\right)(\rho_{Ly+1}\left(y^{*}\left(k+1\right)-y\left(k\right)-\sum_{i=1}^{Ly}\rho_{i}\left(k\right)\hat{\phi_{l}}\left(k\right)\Delta y\left(k-i+1\right)\right)}{{\left(\lambda +{\hat{\phi }}_{Ly+1}{\left(k\right)}^{2}\right)}^{2}}\\ \; & -\frac{\left(\lambda -{\hat{\phi }}_{Ly+1}{\left(k\right)}^{2}\right)\sum_{i=Ly+2}^{Ly+Lu}\rho_{i}\hat{\phi_{l}}\left(k\right)\Delta u\left(k-Ly-i+1\right))}{{\left(\lambda +{\hat{\phi }}_{Ly+1}{\left(k\right)}^{2}\right)}^{2}},l=Ly+1\\ \; & -\frac{{\hat{\phi }}_{Ly+1}\left(k\right)\sum_{i=Ly+2}^{Ly+Lu}\rho_{i}\Delta u\left(k-Ly-i+1\right)}{\left(\lambda +{\left|{\hat{\phi }}_{Ly+1}\left(k\right)\right|}^{2}\right)},Ly+2\leq l\leq Ly+Lu \end{array}\right.$$

It is worth mentioning that the FNN’s membership function is typically a Gaussian radial basis function with attenuation on both sides and is radially symmetric. It has a significant mapping influence on the input when the selected center is quite close to the query point. As a result, the FNN provides the advantages of a fast convergence and a lower likelihood of falling into the local optimum, making it ideal for real-time PG estimation.

### 3.3. Control Scheme of EFFMFAC

**Figure 6:**
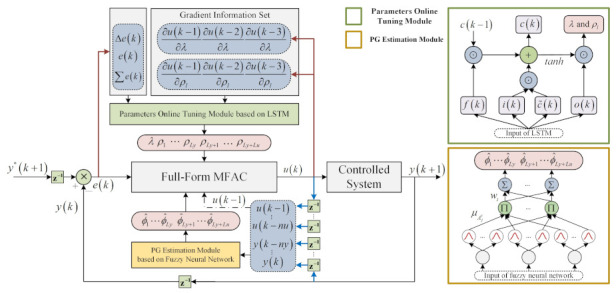
Structure diagram of EFFMFAC.

This study set out to adjust several vital parameters λ and $\rho_{1},\rho_{2},\cdots ,\rho_{Ly+Lu}$ of the FFMFAC online and accurately estimate values in the PG vector, an enhanced FFMFAC is proposed. The general framework is illustrated in Figure 6. The left sub-figure is the general architecture of the proposed algorithm. The upper and lower sub-figures on the right represent the online parameter adjustment module and the PG estimation module. With the current time set to time *k*, EFFMFAC utilizes the current and past I/O information vector of the controlled system as FNNs input and they complete the online estimation of PG values online. The LSTM then takes the set containing the system error information and gradient information as input to perform the online adjustment of vital parameters in FFMFAC. Finally, based on PG estimated values and adjusted parameters, the input signal u(k) and the output y(k+1) are obtained:

In general, the control scheme of EFFMFAC is established as below:

- Step1. PG values estimation based on FNNs:

(52) $$\hat{\phi }\left(k\right)=\sum_{i=1}^{n}\omega^{i}\left(p_{0}^{i}+p_{1}^{i}x_{1}+\cdots +p_{num}^{i}x_{num}\right)/\sum_{i=1}^{n}\omega^{i}$$

(53) $${\hat{\phi }}_{f,Ly,Lu}\left(k\right)=\left[{\hat{\phi }}_{1}\left(k\right),\cdots ,{\hat{\phi }}_{Ly}\left(k\right),\cdots ,{\hat{\phi }}_{Ly+Lu}\left(k\right)\right]$$

- Step2. Vital parameters’ online adjustment based on LSTMs:

(54) $$out_{l}\left(k\right)=\sigma \left(w_{out}h\left(x_{\mathrm{lstm}}\left(k\right)\right)+b_{lstm}\right)$$

(55) $$\begin{array}{c} \lambda =out_{l1}\left(k\right)\\ \rho_{l}=out_{l(l+1)}\left(k\right),l=1,2,\cdots ,Ly+Lu \end{array}$$

- Step3. Control scheme of the enhanced FFMFAC:

(56) $$\begin{array}{cc} u(k)= & u\left(k-1\right)+\frac{\rho_{Ly+1}{\hat{\phi }}_{Ly+1}\left(k\right)\left(y^{*}\left(k+1\right)-y\left(k\right)\right)}{\lambda +{\left|{\hat{\phi }}_{Ly+1}\left(k\right)\right|}^{2}}\\ \; & -\frac{{\hat{\phi }}_{Ly+1}\left(k\right)\sum_{i=1}^{Ly}\rho_{i}{\hat{\phi }}_{i}\left(k\right)\Delta y\left(k-i+1\right)}{\lambda +{\left|{\hat{\phi }}_{Ly+1}\left(k\right)\right|}^{2}}\\ \; & -\frac{{\hat{\phi }}_{Ly+1}\left(k\right)\sum_{i=Ly+2}^{Ly+Lu}\lambda {\hat{\phi }}_{i}\left(k\right)\Delta u\left(k-Ly-i+1\right)}{\lambda +{\left|{\hat{\phi }}_{Ly+1}\left(k\right)\right|}^{2}} \end{array}$$

(57) $$H_{Ly,Lu}\left(k\right)={\left[y\left(k\right),\cdots ,y\left(k-Ly+1\right),u\left(k\right),\cdots ,u\left(k-Lu+1\right)\right]}^{T}$$

(58) $$y\left(k+1\right)=y\left(k\right)+\phi_{f,Ly,Lu}^{T}\left(k\right)\Delta H_{Ly,Lu}\left(k\right)$$

- Step4. Weight coefficients update calculation:

(59) $$w_{fnn}\left(k+1\right)=w_{fnn}\left(k\right)-\beta \frac{\partial J}{\partial w_{fnn}\left(k\right)}+\alpha \Delta w_{fnn}\left(k\right)$$

(60) $$w_{lstm}\left(k+1\right)=w_{lstm}\left(k\right)-\eta \frac{\partial J}{\partial w_{lstm}}$$

where $w_{fnn}$ and $w_{lstm}$ refer to all weight coefficients to be trained in FNNs and LSTMs, and the specific update formulas of all weight coefficients—omitted here for the sake of brevity—can be found in Section 3.1 and Section 3.2.

## 4. Simulation and Experimental Results

In the experimental part, a single-input–single-output (SISO) discrete nonlinear system simulation and a three-tank system simulation were carried out to demonstrate the effectiveness and applicability of EFFMFAC. All tested methods involved in these two simulations are FFMFAC [38], PSO-based FFMFAC [25], BP-based FFMFAC [24], RBF-based FFMFAC [23] and the proposed FFMFAC, which are denoted by the following abbreviations for the sake of brevity: FFMFAC, FFMFAC-PSO, FFMFAC-BP, FFMFAC-RBF and EFFMFAC.

It should be noted that the tracking curves of FFMFAC in two simulations are nearly identical to the corresponding tracking curves in the cited reference [38], which can be used as a benchmark to demonstrate the superiority of EFFMFAC.

### 4.1. SISO Discrete Nonlinear System Simulation

The SISO discrete nonlinear system is expressed as [38]:

(61) $$\begin{array}{cc} y(k+1)= & \frac{2.5y(k)y(k-1)}{1+y^{2}\left(k\right)+y^{2}\left(k-1\right)}+1.2u\left(k\right)+0.09u\left(k\right)u\left(k-1\right)+1.6u\left(k-2\right)\\ \; & +0.7\sin(0.5(y(k)+y(k-1)))\cos(0.5(y(k)+y(k-1))) \end{array}$$

The system desired output is expressed as

(62) $$y^{*}\left(k+1\right)=5\sin\left(k\pi /50\right)+2\cos\left(k\pi /20\right)$$

The initial parameters of EFFMFAC are set as listed in Table 1. The control output linearization constant Ly is 1, and the control input linearization constant Lu is 2, implying that there are three PG values to estimate and four parameters (λ, $\rho_{1}$, $\rho_{2}$ and $\rho_{3}$) to adjust online at each time step. The initial parameter selection in FFMFAC in this simulation is consistent with that in the cited reference [38]. The parameters in the neural networks are determined by the grid search method [39] to ensure that EFFMFAC can achieve the best control performance.

- Tracking performance of all methods

**Figure 7:**
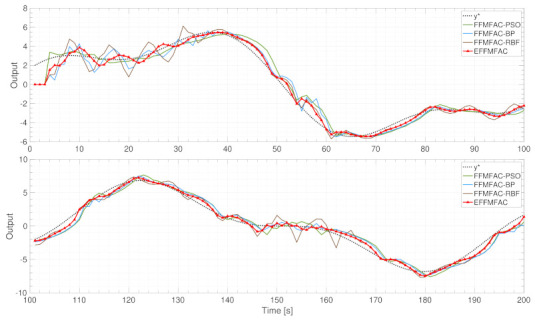
Tracking curves of all methods.

Figure 7 illustrates the tracking performance of all algorithms including EFFMFAC. To clearly illustrate the dynamic properties of tracking curves, the time axis is divided in half and presented separately to better compare the tracking performances of different methods. In terms of overall tracking performance, all tracking curves first fluctuate to varied degrees before progressively stabilizing. Among these methods, EFFMFAC performs best. Specifically, the tracking curve of EFFMFAC has relatively tiny fluctuations in the first 30 s, and it can track the target curve well after 30 s, and its degree of fit to the target curve $y^{*}$ is the best.

FFMFAC-PSO performs better than EFFMFAC in the first 30 s, but its subsequent tracking error is much bigger than that of EFFMFAC. The primary reason for this is because in the early stages, the PSO method’s powerful search capability can identify more appropriate parameters, resulting in a higher initial tracking performance for the FFMFAC-PSO. However, as the tracking curve stabilizes, the nonlinear approximation capacity of PSO is not as good as that of neural networks, which leads to a decline in its tracking performance.

The tracking performance of FFMFAC-BP and FFMFAC-RBF similarly have a worse tracking performance than EFFMFAC, particularly during the first 40 s, and their tracking curves have pronounced fluctuations, and the degree of fitting to the target curve after that is not as good as EFFMFAC. Especially for FFMFAC-RBF, its curve fluctuations in the two time periods of [0,35] and [145,165] are the largest.

- Ablation analysis

**Figure 8:**
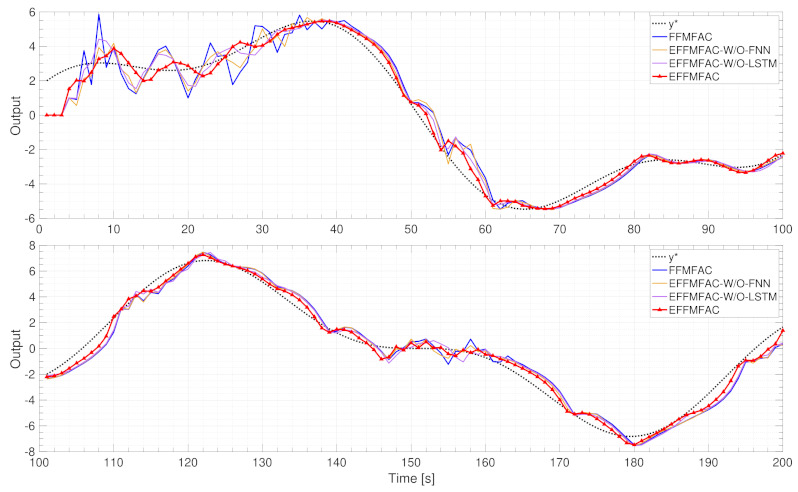
Tracking curves of EFFMFAC and its variants.

To demonstrate the efficiency of the online adjustment module for important parameters and the module for estimating PG values, an ablation analysis of the proposed EFFMFAC is performed, and two temporary methods are introduced as comparison methods. EFFMFAC-W/O-LSTM is a variant of EFFMFAC without the parameters adjustment module, and EFFMFAC-W/O-FNN is a variant of EFFMFAC without the PG estimation module. Together with the original FFMFAC, the tracking curves of these four methods are illustrated in Figure 8.

As illustrated in Figure 8, the tracking performance of FFMFAC is the worst, and its tracking curve fluctuates the most in the first 40 s. EFFMFAC-W/O-LSTM and EFFMFAC-W/O-FNN achieve better tracking performance than FFMFAC, as mainly reflected in the minor fluctuations in the tracking curve at the beginning. The difference in tracking performance illustrates the effectiveness of the online parameter adjustment module and PG values estimation module in the EFFMFAC. Compared with EFFMFAC-W/O-LSTM and EFFMFAC-W/O-FNN, EFFMFAC has an improvement in tracking performance, and its tracking curve fits the target curve best, which proves the correctness of FNNs and LSTMs, implying that the joining of two modules can result in improved control performance.

- Vital parameters’ online adjustment results

**Figure 9:**
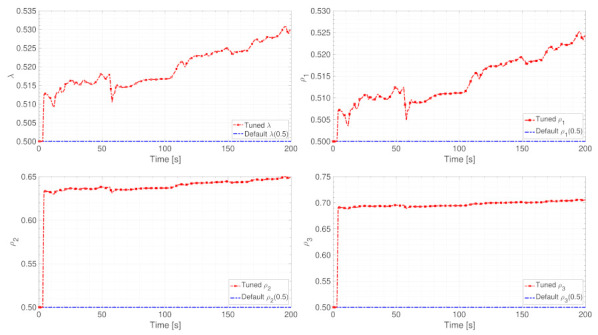
The value curves of the adjustment of parameters λ and ρ.

The online adjustment results of parameters λ and $\rho_{1},\rho_{2},\rho_{3}$ are shown in Figure 9. As illustrated in these four sub-figures, the EFFMFAC can sensitively adjust these vital parameters in real time. In addition, the adjusted parameter values are in the same order of magnitude as the default parameter values, and the difference between the values is small, ensuring the validity of parameters’ online adjustment. In conjunction with the tracking curves in Figure 8, sensitive online parameter adjustment can improve the tracking performance, proving the necessity of the online parameter adjustment and the superiority of LSTM. Furthermore, the value curves of parameters λ and $\rho_{1},\rho_{2},\rho_{3}$ exhibit similarity, which can be explained with the theoretical analysis combined with this simulation. As presented in the control scheme (14), which can be converted as below:

(63) $$\begin{array}{cc} \Delta u(k)= & \frac{\rho_{Ly+1}{\hat{\phi }}_{Ly+1}\left(k\right)\left(y^{*}\left(k+1\right)-y\left(k\right)\right)}{\lambda +{\left|{\hat{\phi }}_{Ly+1}\left(k\right)\right|}^{2}}\\ \; & -\frac{{\hat{\phi }}_{Ly+1}\left(k\right)\sum_{i=1}^{Ly}\rho_{i}{\hat{\phi }}_{i}\left(k\right)\Delta y\left(k-i+1\right)}{\lambda +{\left|{\hat{\phi }}_{Ly+1}\left(k\right)\right|}^{2}}\\ \; & -\frac{{\hat{\phi }}_{Ly+1}\left(k\right)\sum_{i=Ly+2}^{Ly+Lu}\lambda {\hat{\phi }}_{i}\left(k\right)\Delta u\left(k-Ly-i+1\right)}{\lambda +{\left|{\hat{\phi }}_{Ly+1}\left(k\right)\right|}^{2}}\\ = & \frac{\rho_{2}}{\lambda +{\left|{\hat{\phi }}_{2}\left(k\right)\right|}^{2}}{\hat{\phi }}_{2}\left(k\right)\left(y^{*}\left(k+1\right)-y\left(k\right)\right)\\ \; & -\frac{\rho_{1}}{\lambda +{\left|{\hat{\phi }}_{2}\left(k\right)\right|}^{2}}{\hat{\phi }}_{2}\left(k\right){\hat{\phi }}_{1}\left(k\right)\Delta y\left(k\right)\\ \; & -\frac{\rho_{3}}{\lambda +{\left|{\hat{\phi }}_{2}\left(k\right)\right|}^{2}}{\hat{\phi }}_{2}\left(k\right){\hat{\phi }}_{3}\left(k\right)\Delta u\left(k-3\right) \end{array}$$

where λ and $\rho_{1},\rho_{2},\rho_{3}$ are used to guarantee the smoothness of input u(k). Since the PG value ${\hat{\phi }}_{i}\left(k\right)$ does not significantly change, parameters λ and $\rho_{1},\rho_{2},\rho_{3}$ are required to play a vital role in keeping the term $\rho_{i}\phi_{2}\left(k\right)/\left(\lambda +{\left|\phi_{2}\left(k\right)\right|}^{2}\right),i=1,2,3$ from excessively changing. As a result, the value curves of λ and $\rho_{i}$ have comparable tendencies.

- PG estimation results

**Figure 10:**
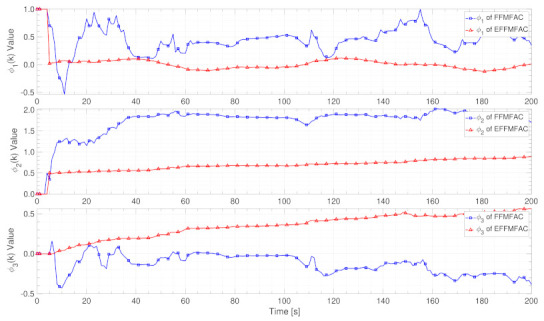
PG estimated value curves.

Figure 10 shows the PG estimated value curves of FFMFAC and EFFMFAC. Three PG estimated value curves of FFMFAC fluctuate wildly, whereas the three PG estimated value curves of EFFMFAC are much flatter, indicating that the dynamics of FFMFAC’s PG are so complicated that its projection estimation algorithm is unable to accurately estimate its actual value. In conjunction with Figure 8, EFFMFAC achieves better tracking performance than FFMFAC, reflecting the validity of the PG estimation module and the superiority of FNNs.

- Parameter sensitivity analysis

**Figure 11:**
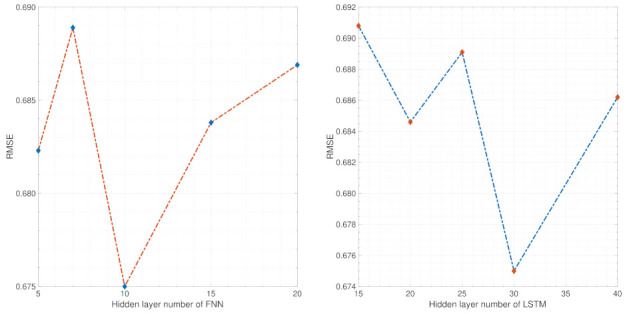
Parameter sensitivity analysis of the introduced neural networks’ hidden layers number.

The parameter sensitivity analysis of EFFMFAC is performed in this simulation under the univariate setting. As shown in Figure 11, the left sub-figure depicts the sensitivity analysis of FNNs’ hidden layers number. Different numbers can influence the RMSE result, and an ideal control performance can be achieved when the number of hidden layers is approximately 10. The right sub-figure shows the sensitivity analysis of the LSTM hidden layers number. Similarly, different numbers affect the control performance and the lowest RMSE result is obtained when the number is approximately 30. The above parameter sensitivity analysis supports the rationality of the initialization work in Table 1.

To briefly summarize, in this simulation, all of the figures illustrated above indicate that the proposed algorithm can accurately estimate PG values and sensitively adjust vital parameters λ, $\rho_{1}$, $\rho_{2}$ and $\rho_{3}$ online. By comparing with other cited methods and variants of EFFMFAC, it can be found that EFFMFAC achieves the best control performance. The rationality of the two modules’ PG estimation module and parameter adjustment module, as well as the superiority of introduced neural networks have been demonstrated.

### 4.2. Three-Tank System Simulation

**Figure 12:**
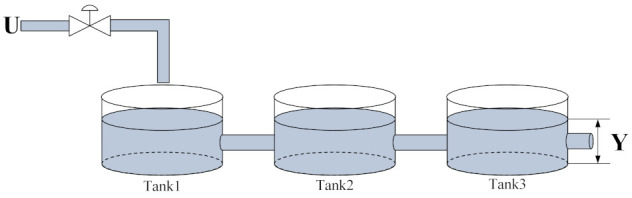
Structure diagram of the three-tank system.

The three-tank system [40] is a typical nonlinear and time-delayed system. As illustrated in Figure 12, this system is comprised of three identical cylindrical tanks, which are connected to each other through cylindrical pipes. The output *Y* (cm) is the liquid level of Tank3, while the control input *U* is the flow opening (%) into the tank.

In the simple three-tank system, the transfer function of the output *Y* and control input *U* is determined as follows:

(64) $$G\left(s\right)=\frac{Y(s)}{U(s)}=\frac{Ke^{-\tau s}}{\left(T_{1}s+1\right)\left(T_{2}s+1\right)\left(T_{3}s+1\right)}$$

where *K* is the system gain, τ is the delay factor and T1, T2 and T3 are time constants. In this simulation, the values of the aforementioned parameters are as follows:

(65) $$\begin{array}{c} \left[K\;\tau \;T_{1}\;T_{2}\;T_{3}\right]=\;\left\{\begin{array}{cc} \left[4.5\;24\;8\;8\;8\right] & k<400\\ \left[5\;24\;8\;8\;8\right] & 400\leq k<800\\ \left[5\;40\;6\;6\;6\right] & 800\leq k<1000 \end{array}\right. \end{array}$$

With the transfer function (64) and selected parameters (65), the three-tank system can be determined:

(66) $$\begin{array}{c} y\left(k+1\right)=\;\left\{\begin{array}{c} 2.6475y(k)-2.3364y(k-1)+0.6873y(k-2)\;\\ +0.001334u(k-24)+0.00486u(k-25)\;+0.001106u(k-26),k<400\;\\ 2.6475y(k)-2.3364y(k-1)+0.6873y(k-2)\;\\ +0.001482u(k-24)+0.0054u(k-25)\;+0.001229u(k-26),400\leq k<800\;\\ 2.5394y(k)-2.1496y(k-1)+0.6065y(k-2)\;\\ +0.003406u(k-40)+0.01203u(k-41)\;+0.0026u(k-42),800\leq k<1000 \end{array}\right. \end{array}$$

The desired value of the system output is as follows:

(67) $$y^{*}\left(k\right)=10$$

The initial parameters in this simulation are set as listed in Table 1. The control output linearization constant Ly is 1, and the control input linearization constant Lu is 2, implying that there are three PG values to estimate and four parameters (λ, $\rho_{1}$, $\rho_{2}$ and $\rho_{3}$) to adjust online at each time step. Similarly to the first simulation, the initial parameter selection in FFMFAC in this simulation is consistent with that in the cited reference [40].

- Tracking performance of all methods

**Figure 13:**
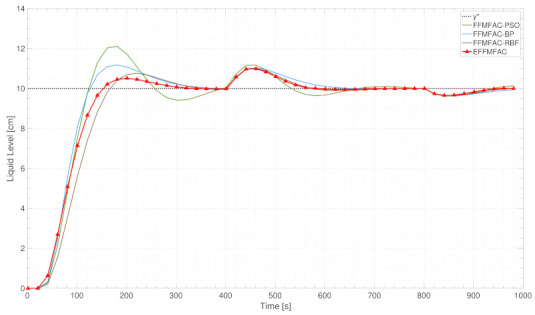
Tracking curves of cited methods and EFFMFAC.

Figure 13 compares the proposed EFFMFAC with other cited methods. In terms of overall tracking performance, EFFMFAC outperforms all others, rapidly and steadily tracking the target curve. From 0 to 400 s, although the increasing time of EFFMFAC is longer than that of FFMFAC-PSO and FFMFAC-BP, the fluctuation of EFFMFAC is the smallest and it is the first to reach a steady state, while the other three algorithms have not yet stabilized. From 400 to 800 s, the tracking curves of EFFMFAC and FFMFAC-RBF are very close, and both track the target curve stably after 600 s, whilst the remaining two methods stabilize after 680 s. In the last 200 s, the tracking curves of the four methods are relatively close, and the tracking error of EFFMFAC is slightly smaller than that of the other three. Figure 13 depicts the EFFMFAC’s superiority in terms of tracking performance, implying the effectiveness of incorporated neural networks.

- Ablation analysis

**Figure 14:**
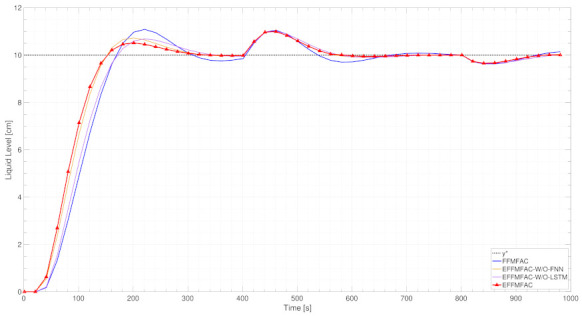
Tracking curves of EFFMFAC and its variants.

Aiming to prove the validity of vital parameters’ online adjustment and PG values estimation in this simulation, ablation analysis was carried out. Similarly to the SISO discrete nonlinear system simulation, two temporary EFFMFAC-W/O-LSTM and EFFMFAC were introduced as comparison methods. The tracking curves for these four methods, together with the original FFMFAC, are displayed in Figure 14.

As illustrated in Figure 14, the tracking performance of FFMFAC is inferior to that of other methods. Compared with the other three tested algorithms, has the most considerable fluctuations and is unable to maintain consistent tracking of the target curve over time. EFFMFAC-W/O-LSTM and EFFMFAC-W/O-FNN both outperform FFMFAC in terms of tracking performance, demonstrating the efficiency of the EFFMFAC’s online parameter adjustment and PG value estimation modules. It should be noted that the tracking performance of EFFMFAC-W/O-LSTM is not as good as that of EFFMFAC-W/O-FNN. Compared with EFFMFAC-W/O-LSTM and EFFMFAC-W/O-FNN, EFFMFAC has improved tracking performance, and its tracking curve has the shortest rise time and is the fastest to reach a steady-state, demonstrating the reasonableness of optimizing FFMFAC by cooperating the two modules based on neural networks.

- Vital parameters’ online adjustment results

**Figure 15:**
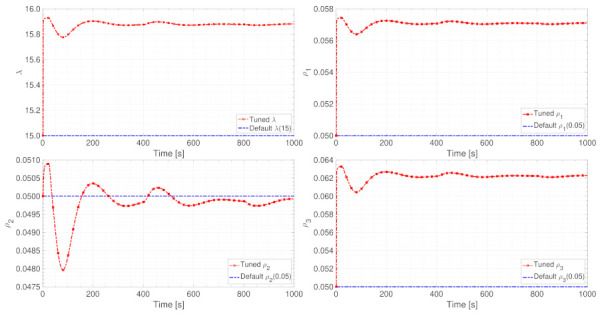
Parameter adjustment value curves of λ and ρ.

The results of the online adjustment of parameters λ and $\rho_{1},\rho_{2},\rho_{3}$ are shown in Figure 15. As illustrated in these four sub-figures, the EFFMFAC can sensitively adjust these vital parameters in real-time. In addition, the adjusted parameter values are in the same order of magnitude as the default parameter values, and the difference between the values is small, ensuring the validity of online parameters adjustment.

- PG estimation results

**Figure 16:**
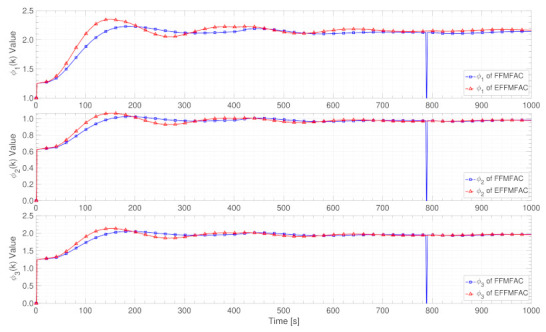
PG estimated value curves.

Figure 16 shows the PG estimated value curves of FFMFAC and EFFMFAC. The fluctuations of these two methods’ PG value curves are relatively small, and their PG estimated values are also very close. The only thing to note is that the default projection estimation method in FFMFAC triggered the reset mechanism at the 789 s, while the FNN-based PG estimation algorithm can always perform estimation calculations, which shows the effectiveness of FNNs. Compared with Figure 14, the similarity of the PG value curves of these two PG estimation methods can explain that the optimization performance of PG estimation based on FNNs is not as good as parameter adjustment.

- Parameter sensitivity analysis

**Figure 17:**
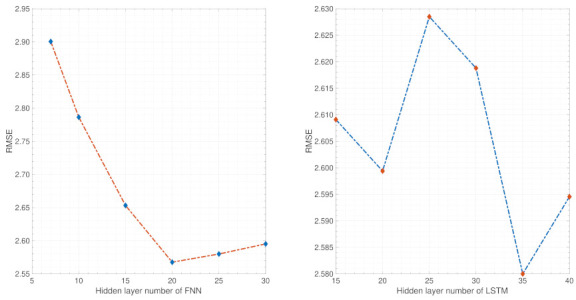
Parameter sensitivity analysis of introduced neural networks hidden layers number.

The parameter sensitivity analysis of EFFMFAC is performed in a three-water tank simulation under the univariate setting. As shown in Figure 17, the left sub-figure demonstrates the sensitivity analysis of FNNs’ hidden layers number. It can be found that insufficient hidden layers may lead to a decrease in the control performance, and an ideal control performance can be achieved when the number of hidden layers is approximately 20. The right sub-figure shows the sensitivity analysis of the LSTM hidden layers number. Similarly, different numbers influence the RMSE result, and the best RMSE result is obtained when the number is approximately 35. The above parameter sensitivity analysis supports the rationality of the initialization work in Table 1.

In general, the EFFMFAC outperforms all other tested methods in this three-tank system simulation. All of the figures illustrated above can prove that both the FNN-based PG estimation module and the LSTM-based online parameter adjustment module can optimize FFMFAC, implying the effectiveness of all introduced neural networks. In addition, the optimization performance of PG estimation is not as good as parameter adjustment work, showing that the parameter adjustment has a more significant impact on the control performance in this simulation.

### 4.3. Simulation Results and Analysis

Five individual metrics are provided to more completely evaluate EFFMFAC’s control performance, namely the root mean square error (RMSE), the integral absolute error (IAE), the integral absolute variation of the control signal (IAVU), the maximum overshoot (MO) and the imprecise control ratio (ICR). These five indices are expressed in (68)–(72) below:

(68) $$RMSE=\sqrt{\frac{1}{N}\sum_{k=1}^{N}e{\left(k\right)}^{2}}$$

(69) $$IAE=\int_{0}^{t}\left|e_{i}\left(t\right)\right|dt$$

(70) $$IAVU=\int_{0}^{t}\left|\frac{du(t)}{dt}\right|dt$$

(71) $$MO=\max\left(\left(y\left(1\right)-y^{*}\left(1\right)\right),\cdots ,\left(y\left(N\right)-y^{*}\left(N\right)\right)\right)$$

(72) $$\begin{array}{c} ICR\left(\xi \right)=\frac{1}{N}\sum_{k=1}^{N}IC\left(k,\xi \right)\;\\ IC\left(k,\xi \right)=\left\{\begin{array}{cc} 0 & when\left|y\left(k\right)-y^{*}\left(k\right)\right|<\xi \\ 1 & when\left|y\left(k\right)-y^{*}\left(k\right)\right|\geq \xi \end{array}\right. \end{array}$$

The first two indices RMSE and IAE are introduced to evaluate tracking the accuracy of the method, the IAVU is used to evaluate the stability of the control input, the MO is used to evaluate the tracking instability and the ICR is introduced to calculate the time proportion of imprecise control.

#### 4.3.1. Analysis of SISO Discrete Nonlinear System Simulation Results

According to the experimental results listed in Table 2, FFMFAC performs poorly on a variety of indices. FFMFAC-PSO, FFMFAC-BP and FFMFAC-RBF introduce different optimization methods to optimize FFMFAC. From the evaluation results in Table 2, it is obvious that the tracking performance of the above three methods has improved to varying degrees.

Regarding the FFMFAC-BP and EFFMFAC-W/O-FNN, both methods perform the online parameter adjustment of the FFMFAC. EFFMFAC-W/O-FNN has better simulation results in all indices than FFMFAC-BP, and the five indicators are reduced by 4.53%, 4.26%, 4.35%, 8.28% and 1.65%, respectively, which reflects the effectiveness of the gate mechanism of LSTMs. In addition, for FFMFAC-RBF and EFFMFAC-W/O-LSTM, both methods perform the PG estimation of the FFMFAC. Similarly, EFFMFAC-W/O-LSTM performs better than FFMFAC-RBF, and the five indicators are reduced by 9.61%, 11.03%, 40.41%, 1.85% and 1.09%, respectively. Given that FNNs possesses both the local approximation capability of RBF neural networks and the ability to reason adaptively, this explains why FNNs outperform RBF neural networks in PG estimation.

From the simulation results of the EFFMFAC, it can be found that EFFMFAC achieves the best results in various indices. Compared with FFMFAC, it has reduced by 21.69%, 23.43%, 36.31%, 42.55% and 3.26% in most indices, reflecting the superiority of tracking performance. In addition, EFFMFAC also occupies an advantage in all indices compared to its variants EFFMFAC-W/O-FNN and EFFMFAC-W/O-LSTM, which shows the effectiveness of introduced modules.

#### 4.3.2. Analysis of Three-Tank System Simulation Results

According to the experimental results listed in Table 3, the gap between the indicators of all algorithms is relatively small. FFMFAC has the worst performance on RMSE, IAE and ICR, while FFMFAC-PSO has the worst performance on IAVU and MO. Compared with FFMFAC, the tracking performance of FFMFAC-BP has improved in all indicators except MO, and FFMFAC-RBF has achieved better results in all indices.

Regarding FFMFAC-BP and EFFMFAC-W/O-FNN, EFFMFAC-W/O-FNN has better simulation results than FFMFAC-BP in all indices, and the five indicators are reduced by 1.96%, 1.67%, 6.62%, 13.38% and 18.47%, respectively, which reflects that LSTMs perform better than BP neural networks in parameter adjustment. In addition, for the FFMFAC-RBF and EFFMFAC-W/O-LSTM, EFFMFAC-W/O-LSTM performs better than FFMFAC-RBF and the five indicators are reduced by 4.25%, 0.42%, 1.18%, 0.98% and 5.56%, respectively, proving the superiority of FNNs. It is worth noting that the difference between the optimization performance of the FNN-based PG estimation module and LSTMs-based parameter adjustment module on FFMFAC is noticeable, and EFFMFAC-W/O-FNN has reduced by 7.73%, 10.34%, 0.70% and 7.91% in most indices except MO compared to EFFMFAC-W/O-LSTM. It shows that in this simulation, the optimization performance of the parameter adjustment module is better than that of the PG estimation module, and similar results are also reflected in FFMFAC-BP and FFMFAC-RBF.

From the experimental results of EFFMFAC, it can be found that EFFMFAC achieves the best results in various indices. Compared with FFMFAC, it has reduced by 11.21%, 22.02%, 14.17%, 6.71% and 28.27% in all indices, reflecting the superiority of its tracking performance. Additionally, EFFMFAC also occupies an advantage in all indices compared to its variants EFFMFAC-W/O-FNN and EFFMFAC-W/O-LSTM, which shows the effectiveness of introduced modules.

Notably, Table 2 and Table 3 provide the average calculation time for each method at each time step. Although the calculation time of our proposed EFFMFAC is longer than that of other algorithms except FFMFAC-PSO, the EFFMFAC’s average running time is quite fast in comparison to the 1000 ms sampling period, which enables the desired real-time tracking.

Generally speaking, some cited methods only outperform FFMFAC in a few indices, implying that the optimization performance is insufficient and demonstrating the significance of introducing multiple indices to evaluate the control performance of each method. Furthermore, the optimization effects of the introduced neural networks are demonstrated via ablation analysis. As a result, EFFMFAC significantly improves all indices, demonstrating the rationality of algorithm design.

## 5. Conclusions

In this study with the objective of performing the sensitive online adjustment of FFMFAC parameters as well as improving the accuracy of PG estimation, this paper proposes the EFFMFAC for a class of SISO discrete-time nonlinear systems. The significance and novelty of this study lies in the use of LSTMs to sensitively adjust vital parameters λ and $\rho_{1},\rho_{2},\cdots ,\rho_{Ly+Lu}$ online and introducing FNNs to complete PG estimation work in real time, thus dramatically improving the control performance of FFMFAC. In the experimental part, SISO discrete nonlinear system simulation and three-tank system simulation were carried out to verify the validity and superiority of EFFMFAC, and five evaluation indices were provided to evaluate EFFMFAC. The experimental results demonstrated that EFFMFAC achieves the best tracking performance and achieves the best results across all evaluation indices. Previous theoretical results did not include this joint optimization method. EFFMFAC will be applied to MIMO nonlinear systems such as a continuous stirring reactor, distillation tower and vapor compression refrigeration system in subsequent research work to verify its effectiveness.

A major limitation of EFFMFAC lies in the initialization work. Certain neural networks have many initial parameters and need to be adjusted in advance, as inappropriate parameters will affect the tracking performance of the algorithm. Furthermore, although EFFMFAC has better tracking performance than the FFMFAC, it still has potential for optimization. As shown in Figure 8, the tracking error is not reduced in some time periods. A reasonable explanation is that the system output at the next time step may also be related to the system output tracking error in the sliding window. As a result, the optimization of the full-form dynamic linearization method will be part of future research, and the optimized full-form dynamic linearization model does not only consider the changes in the previous input and output of the controlled system but also considers the changes in the output tracking error within a time sliding window [38], which can better represent the controlled system’s complicated dynamic properties.

In the actual complex manufacturing process such as the oil refining production process, chemical production process, etc., the actual output of the system needs to be measured. During the measurement process, disturbance signals will be generated due to the influence of the external environment or sensors, and the measurement noise of the data is an unavoidable issue. EFFMFAC in this paper can be regarded as a pure data-driven control method, as it has not been evaluated in a real-world industrial scene with issues such as the measurement noise and control saturation. To deal with disturbance factors, denoising approaches such as the wavelet threshold denoising method [41] will be implemented into the FFMFAC in future research. Investigating these aspects in the real manufacturing process is critical for practical engineering.

## Figures and Tables

**Figure 4:**
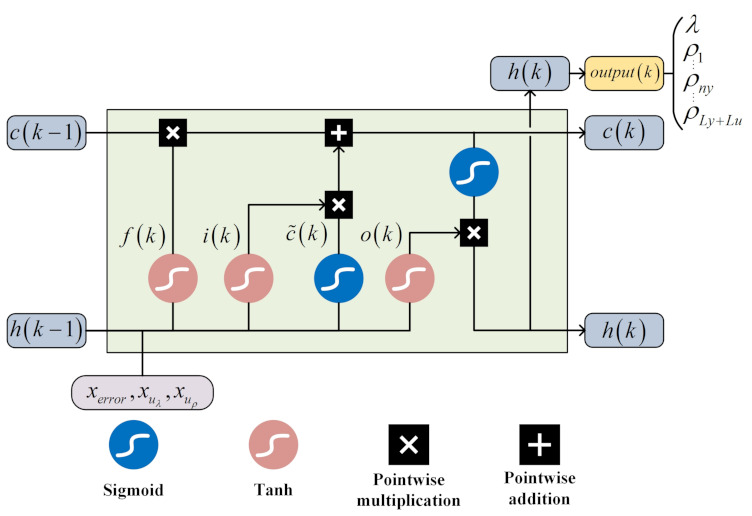
Architecture of the online parameter adjustment module.

**Table 1:** Initialization in two simulations.

SISO Discrete Nonlinear System Simulation		Three-Tank System Simulation	
Initialization Parameter	Value	Initialization Parameter	Value
y(k),k=1,2,3	0	y(k),k=1,2,3	0
u(k),k=1,2,3	0	u(k),k=1,2,⋯,43	0
Ly	1	Ly	1
Lu	2	Lu	2
μ	1	μ	1
ε	$10^{-5}$	ε	$10^{-5}$
${\hat{\phi }}_{f,Ly,Lu}\left(1\right)$	[1 0 0]	${\hat{\phi }}_{f,Ly,Lu}\left(1\right)$	[1 0 0]
λ	0.5	λ	15
$\rho_{l},l=1,2,\cdots ,Ly+Lu$	0.5	$\rho_{l},l=1,2,\cdots ,Ly+L$u	0.05
FNN layers number	5-10-3	FNN layers number	7-20-3
LSTM layers number	15-30-4	LSTM layers number	15-35-4
β	0.5	β	0.2
α	0.02	α	0.01
η	0.05	η	0.02

**Table 2:** Experimental results of SISO discrete nonlinear system simulation.

	*RMSE*	*IAE*	*IAVU*	*MO*	*ICR* (0.1)	AVG Time (ms)
FFMFAC	0.862	135.315	83.088	2.825	0.920	0.51
FFMFAC-PSO	0.784	122.162	47.595	1.821	0.915	80.78
FFMFAC-BP	0.816	126.389	70.768	2.425	0.905	1.06
FFMFAC-RBF	0.812	127.986	92.192	1.726	0.915	0.67
EFFMFAC-W/O-FNN	0.779	120.998	67.304	2.224	0.890	3.68
EFFMFAC-W/O-LSTM	0.734	113.860	54.939	1.694	0.905	0.64
**EFFMFAC**	**0.675**	**103.613**	**52.915**	**1.623**	**0.890**	**3.92**

**Table 3:** Experimental results of three-tank system simulation.

	*RMSE*	*IAE*	*IAVU*	*MO*	*ICR*(0.1)	AVG Time (ms)
FFMFAC	2.899	1280.841	16.455	1.088	0.750	0.16
FFMFAC-PSO	2.689	1204.468	19.717	2.147	0.747	41.29
FFMFAC-BP	2.644	1076.396	15.171	1.182	0.671	0.23
FFMFAC-RBF	2.821	1186.620	14.642	1.017	0.629	0.29
EFFMFAC-W/O-FNN	2.592	1058.998	14.368	1.022	0.547	1.04
EFFMFAC-W/O-LSTM	2.809	1181.162	14.469	1.016	0.594	0.32
**EFFMFAC**	**2.580**	**998.860**	**14.123**	**1.015**	**0.538**	**3.26**

## Data Availability

Not applicable.

## Abbreviations

The following abbreviations are used in this manuscript:

MFAC	Model-free adaptive controller
LSTM	Long short-term memory
FFDL	Full-form dynamic linearization
PG	Pseudo gradient
DDC	Data-driven control
SISO	Single-input–single-output
